# Synergistic effects of glucose tolerance and BMI on cardiovascular events and all-cause mortality in a healthy population: CA.ME.LI.A study 7 years follow-up

**DOI:** 10.1152/ajpendo.00181.2024

**Published:** 2024-08-28

**Authors:** Monica Bignotto, Elena Bianco, Lucia Centofanti, Antonio Russo, Michele Dei Cas, Paola Zermiani, Camillo Morano, Federica Samartin, Emanuela Bertolini, Francesco Bifari, Cesare Berra, Massimo Zuin, Rita Paroni, Pier Maria Battezzati, Franco Folli

**Affiliations:** ^1^Liver and Gastroenterology Unit, Department of Health Sciences, Università degli Studi di Milano, Milan, Italy; ^2^Medicine and Liver Unit, ASST Santi Paolo e Carlo, Milan, Italy; ^3^Clinical Biochemistry and Mass Spectrometry Unit, Department of Health Sciences, Università degli Studi di Milano, Milan, Italy; ^4^Epidemiology Unit, Agency for Health Protection of the Metropolitan City of Milan, Milan, Italy; ^5^Laboratory of Cell Metabolism and Regenerative Medicine, Department of Medical Biotechnology and Translational Medicine, Università degli Studi di Milano, LITA, Segrate, Italy; ^6^Dipartimento Endocrino-Metabolico, IRCCS MultiMedica, Milano, Italy; ^7^Departmental Unit for Diabetes and Metabolic Diseases, ASST Santi Paolo e Carlo, Milan, Italy; ^8^Departmental Unit for Diabetes and Metabolic Diseases, ASST Santi Paolo e Carlo, Milan, Italy

**Keywords:** cardiovascular risk, type 1/type 2 diabetes mellitus, epidemiology, impaired fasting glucose, obesity

## Abstract

The CA.ME.LI.A (CArdiovascular risks, MEtabolic syndrome, LIver and Autoimmune disease) epidemiological study was conducted in Abbiategrasso (Milan, Italy) to identify risk factors for metabolic and cardiovascular disease in an apparently healthy population of northern Italy. The population (*n* = 2,545, 1,251 men, 1,254 women) was stratified according to body mass index [normal body weight (NBW): <25 kg/m^2^; overweight-obese (OWO): ≥25 kg/m^2^] and according to fasting blood glucose [normal fasting glucose: <100 mg/dL; impaired fasting glucose (IFG): 100–125 mg/dL; diabetes mellitus (DM): ≥126 mg/dL]. The incidence of cardiovascular (CV) events and overall mortality were studied by the Kaplan–Meier method using the log rank test. Univariate analysis was conducted with time-dependent Cox models. During the 7-yr follow-up period, 80 deaths and 149 CV events occurred. IFG [hazard ratio (HR): 2.81; confidence interval (CI): 1.37–5.77; *P* = 0.005], DM (HR: 4.88; CI: 1.47–16; *P* = 0.010), or OWO (HR: 2.78; CI:1.68–4.59; *P* < 0.001) all produced significant increases in CV events and deaths. In the combination IFG/OWO (HR: 5.51; CI: 3.34–9.08; *P* < 0.001), there was an apparent additive effect of the two conditions, whereas in the combination DM/OWO (HR: 12.71; CI: 7.48–22; *P* < 0.001), there was an apparent multiplicative effect on the risk for CV events and deaths. In males, the DM/NBW group had a higher incidence of cardiovascular events and deaths than the IFG/OWO group. In contrast, in females, the IFG/OWO group had a higher incidence of cardiovascular events and deaths than the DM/NBW group. In women, there was a greater incidence of CV events in the IFG/OWO group (HR: 6.23; CI: 2.88–13; *P* < 0.001) than in men in the same group (HR: 4.27; CI: 2.15–8.47; *P* < 0.001). Consistent with these data, also all-cause mortality was progressively increased by IFG/DM and OWO, with an apparently exponential effect in the combination DM/OWO (HR: 11.78; CI: 6.11–23; *P* < 0.001). IFG/DM and OWO, alone or in combination, had major effects in increasing mortality for all causes and CV events. The relative contributions of hyperglycemia and overweight/obesity on cardiovascular events and deaths were apparently, to a certain extent, sex dependent. Females were more affected by overweight/obesity either alone or combined with IFG, as compared with males.

**NEW & NOTEWORTHY** For the first time, the combined effects of glucose tolerance and BMI have been investigated in an apparently healthy large population sample of a city in the north of Italy. We found that there are synergistic effects of glucose levels with BMI to increase not only cardiovascular events and deaths but also cancer-related deaths and all-cause mortality.

## Graphical abstract

**Figure:**
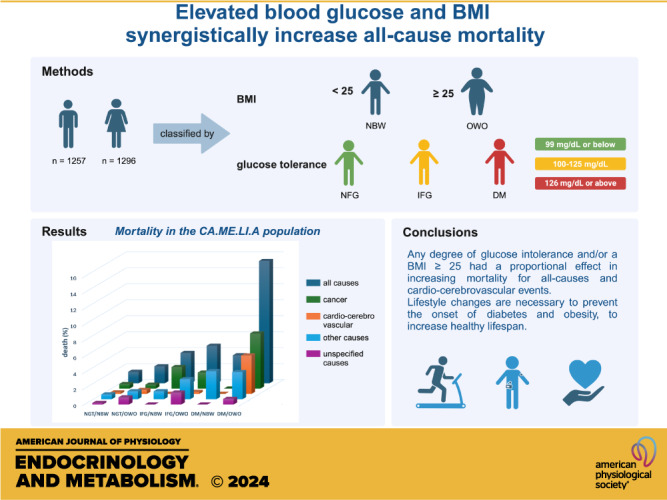

## INTRODUCTION

Obesity and overweight have reached epidemic proportions, and their growth shows no signs of slowing down.

It has been estimated that in 2022, 2.5 billion adults were overweight or obese (1/3 of the entire world), and of these, 890 million (12%) were obese, also affected by associated comorbidities (1). Obesity is associated with a two- to threefold increased risk for death from all causes, including cardiovascular diseases, chronic kidney disease, sleep apnea, cancer, and type 2 diabetes mellitus (T2DM) (2, 3). Of significance, obesity carries also an important psychosocial burden, thus impacting numerous areas of psychosocial functioning (4).

Diabetes mellitus has emerged as one of the most serious and common chronic diseases of our times, causing life-threatening, disabling, and costly complications also significantly reducing life expectancy (5–9). Just over half a billion people are living with diabetes (type 1 or type 2) worldwide, and its prevalence is expected to rise to 12.2% (783.2 million) in 2045 (10). In addition, 541 million people are estimated to be affected by prediabetes, either impaired fasting glucose (IFG), which is characterized by fasting plasma glucose level between 100 and 125 mg/dL, or impaired glucose tolerance (IGT), that is a glucose level between 140 and 200 mg/dL at 2 h after a 75 g glucose tolerance test (10). Type 2 diabetes mellitus can be diagnosed from fasting plasma glucose ≥126 mg/dL (7.0 mmol/L) and/or >200 mg/dL after an oral glucose tolerance test or hemoglobin A1c >6.4% (46 mmol/mol) (11).

In the early 2000s, the term “diabesity” was proposed to indicate the intimate interactive role that obesity has with the development of type 2 diabetes mellitus (12). Chronic subinflammation in the adipose tissue, leading to imbalanced secretion of adipokines, resulting in increased levels of proinflammatory cytokines and decreased levels of anti-inflammatory adipokines, is considered a crucial risk factor for the development of insulin resistance and type 2 diabetes mellitus in obese individuals (13–16).

Individuals with IGT and/or IFG are at higher risk to develop diabetes mellitus and metabolic syndrome X of insulin resistance and to experience cardiovascular (CV) events (myocardial infarction, stroke, CV death) later in life (17–22). Also, obesity is a well-established independent risk factor for development of a variety of cardiovascular diseases (CVD), including heart failure, coronary heart disease, atrial fibrillation, and arterial hypertension (3, 23–25).

CA.ME.LI.A (CArdiovascular risks, MEtabolic syndrome, LIver and Autoimmune disease) is an epidemiological study conducted between 2009 and 2011 in the city of Abbiategrasso (Milan, Italy). The prevalence of cardiovascular risk factors, metabolic syndrome X of insulin resistance, and liver diseases in the CA.ME.LI.A study population has been reported elsewhere (26). Here, we present the findings of the 7-yr follow-up conducted in the CA.ME.LI.A population. The study aims to investigate the impact of hyperglycemia (IFG and DM) and increased body mass (overweight/obesity) on the incidence of cardiovascular events and all-cause mortality, including cardiovascular events (heart and cerebrovascular), cancer, and other diseases.

## MATERIALS AND METHODS

### The CA.ME.LI.A Population

The CA.ME.LI.A study organization, planning, medical examination, and participants’ baseline clinical, ultrasound, and biochemical data have been previously reported (26). The participants’ baseline data were collected between May 2009 and September 2011 (26). The data from the follow-up study period are reported here. All events that occurred from the day of enrollment until August 30, 2017, retrievable in the digital records of the Milan 1 Health District, based on the disease diagnosis coding system of the hospital discharge forms (in Italian: “Scheda Dimissione Ospedaliera” SDO), were considered. The compilation of the “SDO” (hospital discharge form) was based on ICD-9-CM (International Classification of Diseases, 9th Revision, Clinical Modification) codes as previously reported (26). For the present report, causes of death were then coded according to the ICD-10 (International Classification of Diseases, 10th Revision) coding system.

To study the relationships between various degrees of glucose tolerance and overweight/obesity, the population was stratified according to body mass index (BMI) and fasting plasma glucose (3, 27, 28). BMI: *1*) normal body weight subjects (NBW): BMI of <25 kg/m^2^; *2*) overweight and obese subjects (OWO): BMI of ≥25 kg/m^2^. Fasting plasma glucose: *1*) normal fasting glucose subjects (NFG): fasting plasma glucose level of <100 mg/dL; *2*) impaired fasting glucose subjects (IFG): fasting plasma glucose level of 100–125 mg/dL; and *3*) type 2 diabetes mellitus (DM) subjects: fasting plasma glucose level of ≥126 mg/dL.

To verify the potential interaction of BMI with fasting plasma glucose, analyses were performed by considering all possible (six) combinations: *1*) NFG/NBW, *2*) NFG/OWO, *3*) IFG/NBW, *4*) IFG/OWO, *5*) DM/NBW, and *6*) DM/OWO.

### Data Analysis, Visualization, and Interpretation

Data were expressed as counts, percentages, prevalence ratios, and 95% confidence intervals (CIs). Continuous variables are summarized as mean values and standard deviations (SDs). In univariate analyses, the significance level of differences among groups was assessed by the nonparametric Mann–Whitney or Kruskal–Wallis test. To assess the presence of a trend across groups of patients ordered according to their glucose tolerance and BMI levels, the Kruskal–Wallis test was preliminarily performed on the variable of interest, followed, in the case of a significant result, by a nonparametric test for trend based on a method described by Cuzick (29). For categorical variables, the Fisher’s exact test was employed. A modified Poisson approach was used to estimate relative risks, either as a crude value or after covariate adjustment, and their CI and significance levels were assessed using robust error variances.

The study of the incidence of events was carried out using the Kaplan–Meier method, with the log-rank test to assess statistical significance levels. For this study, *time 0* was the date of enrollment in the CA.ME.LI.A study. The predefined final observation dates were considered that of the first cardiovascular event, or the date of death for cardiovascular causes, or the death for all other causes.

For the univariate analysis of the association between any continuous or categorical variable, considered as the risk factor, and the incidence of each outcome event, the aforementioned risk factors were introduced in single-variable, time-dependent Cox models.

To identify the variables retaining an independent prognostic value, those associated (*P* < 0.10) in the univariate analyses with the incidence of an outcome event were studied within the framework of multivariate analysis by means of time-dependent multivariate Cox models.

The study protocol established that, in consideration of the multiple associations between the variables under study and the sex of the subjects, the main analyses were conducted separately in the two sexes. To contextualize the study data, assuming an incidence of events or cardiovascular death of 10% during 7 yr after enrollment, and to demonstrate significance (*P* < 0.05, power of 90%), a difference of ≥5% between the incidence of events in the two groups, it was calculated that 1,189 subjects would be needed using the two-tailed log-rank test (30)_._ In the present study, 1,257 male and 1,297 female subjects were enrolled for all analyses. The level of statistical significance was *P* < 0.05 in two-tailed tests.

Collected data were exported, and statistical analyses were carried out using the Stata software (StataCorp. 2023. Stata Statistical Software: Release 18. College Station, TX: StataCorp LLC).

## RESULTS

The study population included 2,554 subjects (1,257 men and 1,297 women) residing in the city of Abbiategrasso (Milano, Italy). The follow-up period extended from May 2009 to August 30, 2017, with a median duration of 7.4 yr, an interquartile range of 6.8–7.8 yr, and a maximum follow-up duration of 8.3 yr. Overall, the population was followed for 17,776 person-years. During this period, 35 subjects were lost to follow-up.

### Analysis of the Population According to Glucose Tolerance and BMI

The population included 2,554 individuals (51% women and 49% men) aged 18–77 yr, (mean ± SD = all patients: 47.7 ± 14.9 yr; women: 48.0 ± 15.1 yr; men: 47.3 ± 15.1 yr; *P* = 0.221). After stratification in different age-groups (<35, 35–44, 45–54, 55–64, and ≥65 yr), gender distribution did not differ significantly, but a highly significant difference emerged when considering fasting plasma glucose and BMI categories (Supplemental Table S1). Such gender difference was evident in the older age class (≥ 65 yr), in which women were more prevalent. Regarding glucose levels, around two-third (70.4%) of the study participants had NFG, followed by IFG (22.9%) and DM (6.7%) categories (Table 1). Considering body mass index, the population was divided in NBW (48.9%) and OWO (51.1%). Regarding gender distribution, males were more prevalent in the IFG, DM, and OWO categories, whereas females were more prevalent in the NFG and NBW categories (*P* ≤ 0.0001) (Table 1 and Supplemental Fig. S1, *A* and *B*). Participants’ age increased as fasting glucose levels and BMI increased, with some differences in males and females (Supplemental Fig. S1, *C* and *D*).

**Table 1.** Gender distribution of the CA.ME.LI.A population based on glucose tolerance and BMI

	Total Population	Men	Women	*P* Value
*n*	2,553	1,257	1,296*	
NFG	1,796 (70.4%)	770 (61.3%)	1,026 (79.2%)	<0.0001
IFG	585 (22.9%)	377 (30.0%)	208 (16.0%)	
DM	172 (6.7%)	110 (8.7%)	62 (4.8%)	
NBW (BMI of <25 kg/m^2^)	1,250 (48.9%)	492 (39.1%)	758 (58.4%)	<0.0001
OWO (BMI of ≥25 kg/m^2^)	1,304 (51.1%)	765 (60.9%)	539 (41.6%)	

BMI, body mass index; CA.ME.LI.A, CArdiovascular risks, MEtabolic syndrome, LIver and Autoimmune disease; DM, diabetes mellitus; IFG, impaired fasting glucose; NBW, normal body weight; NFG, normal fasting glucose; OWO, overweight-obese.

*One woman lacking glycemic data.

In the population with BMI of <25 kg/m^2^ (NBW), 30%–40% of subjects, in both sexes, were younger than 35 yr. In men with BMI of >25 kg/m^2^ (OWO), the frequency of older subjects progressively increased up to the 45–54 yr class, but it subsequently decreased. Among OWO women, the trend toward increasing frequency of aged subjects was consistent over the whole age class spectrum (Supplemental Fig. S1*D*). The cause of the fluctuating trend of the age distribution in overweight/obese class among men, first “in crescendo” and then “in diminuendo,” could be ascribed possibly to the higher mortality rate of men versus women after 55 yr. In women with BMI of ≥25 kg/m^2^, the trend is opposite to that seen for BMI of <25 kg/m^2^, and the frequency of subjects included in the age categories increased with age.

In the different glucose categories (NFG, IFG, DM), NFG females had a lower BMI than NFG males (*P* ≤ 0.0001) (Supplemental Fig. S2). IFG males and females had similar BMI, whereas DM females had higher BMI than males. Of note, BMI of females showed greater variability than that of males.

### The Interrelationship between Glucose Levels and BMI

To better understand the relationship between impaired fasting glucose and diabetes mellitus (IFG/DM) with overweight/obesity, the CA.ME.LI.A population was stratified into six subgroups, based on fasting glucose levels and BMI. The number of males was higher in the five groups with either increased glucose or increased BMI, whereas in the group with both normal glucose tolerance and normal body weight (NFG/NBW), females were significantly more represented than males (*P* ≤ 0.0001) (Table 2).

**Table 2.** CA.ME.LI.A population stratified by gender, BMI, and glucose tolerance

	Total Population	Men	Women	*P* Value
*n*	2,545*	1,251	1,294	
NFG/NBW	1,039 (40.8%)	372 (29.7%) (14.5%)	667 (51.6%) (26.2%)	<0.0001
NFG/OWO	754 (29.6%)	395 (31.6%) (15.5%)	359 (27.7%) (14.0%)	
IFG/NBW	183 (7.2%)	105 (8.4%) (4.1%)	78 (6.0%) (3.0%)	
IFG/OWO	398 (15.7%)	269 (21.5%) (10.5%)	129 (10.0%) (5.1%)	
DM/NBW	28 (1.1%)	15 (1.2%) (0.5%)	13 (1.0%) (0.5%)	
DM/OWO	143 (5.6%)	95 (7.6%) (3.7%)	48 (1.9%) (3.7%)	

The % was calculated on the total number of subjects in the group (column). For men and women columns, an additional % vs. the whole population is also reported on the left. BMI, body mass index; CA.ME.LI.A, CArdiovascular risks, MEtabolic syndrome, LIver and Autoimmune disease; DM, diabetes mellitus; IFG, impaired fasting glucose; NBW, normal body weight; NFG, normal fasting glucose; OWO, overweight-obese.

*Nine patients of the whole CA.ME.LI.A population were excluded from the analysis for the lack of data, either BMI or glucose tolerance.

Table 3 and Supplemental Table S2 (*a* and *b*) and Supplemental Table S3 show anthropometric, clinical, and laboratory characteristics of the six subgroups in the CA.ME.LI.A population. Mean glucose levels increased in parallel with age. OWO was associated with increased glucose levels in NFG, IFG, and DM, as compared with NBW subjects. Values of insulin and Homeostatic Model Assessment for Insulin Resistance (HOMA-IR) progressively increased going from NFG to IFG and to DM. For each glucose category, i.e., NFG, IFG, and DM, those who had a BMI of <25 kg/m^2^ showed lower insulin and HOMA IR as compared with those with a BMI of ≥25 kg/m^2^. A similar trend was also present for triglyceride levels, with the highest level in DM/OWO subjects. HDL cholesterol showed a trend toward decreasing values with increasing glucose levels, both in NBW and OWO subjects. Incidence of C-reactive protein (CRP) >5 mg/L, of metabolic (dysfunction)-associated fatty liver disease (MAFLD; previously called nonalcoholic fatty liver disease) (31, 32), and of arterial hypertension showed a significant progressive increase from NFG to IFG and to DM. Both conditions were further markedly increased in each class of glucose levels when OWO was also present (all, *P* < 0.0001).

**Table 3.** Clinical and laboratory characteristics of CA.ME.LI.A population stratified by glucose tolerance and BMI

	NFG/NBW	NFG/OWO	IFG/NBW	IFG/OWO	DM/NBW	DM/OWO
*n*	1,039	754	183	398	28	143
Age, yr (*P* ≤ 0.0001)	40.6 ± 13.6	48.9 ± 14	51.6 ± 14.5	55.9 ± 12	56.9 ± 13.1	62.1 ± 9.4
Weight, kg (*P* ≤ 0.0001)	60.9 ± 9.2	80.6 ± 12.7	64.7 ± 9.7	84.4 ± 13.0	63.8 ± 8.3	86.5 ± 16.3
Height, cm (*P* = 0.07)	166.4 ± 9.7	167.3 ± 10.4	168.1 ± 10.0	167.8 ± 10.0	167.4 ± 8.9	165.5 ± 10.4
SBP, mmHg (*P* ≤ 0.0001)	114.1 ± 14.7	125.6 ± 16.1	123.9 ± 17.3	133.1 ± 18.3	125.3 ± 19.1	138.2 ± 19.4
DBP, mmHg (*P* ≤ 0.0001)	72.3 ± 9.5	79.0 ± 9.5	75.7 ± 10.0	82.8 ± 10.1	74.1 ± 9.9	81.1 ± 10.9
Waist circumference, cm (*P* ≤ 0.0001)	81.8 ± 7.8	98.4 ± 9.3	86.1 ± 8.4	102.1 ± 10.3	83.7 ± 8.5	107.5 ± 12.6
Glucose, mg/dL (*P* ≤ 0.0001)	87.9 ± 6.7	90.9 ± 5.6	105.8 ± 6.1	107.9 ± 6.4	143.1 ± 40.5	159.0 ± 49.5
Insulin, U/L (*P* ≤ 0.0001)	4.3 ± 2.1	6.6 ± 3.4	5.7 ± 3.5	9.3 ± 7.4	9.0 ± 7.7	12.4 ± 10.4
HOMA index (*P* ≤ 0.0001)	0.9 ± 0.5	1.5 ± 0.8	1.5 ± 1.0	2.5 ± 2.1	3.1 ± 2.7	5.2 ± 6.1
Total cholesterol, mg/dL (*P* ≤ 0.0001)	198.1 ± 39.1	211.6 ± 40.0	209.0 ± 39.6	211.0 ± 40.8	196.5 ± 34.5	201.1 ± 40.7
HDL cholesterol, mg/dL (*P* ≤ 0.0001)	59.6 ± 13.4	53.0 ± 13.0	58.0 ± 14.1	49.6 ± 11.9	51.5 ± 14.1	46.7 ± 10.5
LDL, mg/dL (*P* ≤ 0.0001)	127.4 ± 30.8	144.0 ± 32.1	137.7 ± 33.2	144.2 ± 32.9	130.8 ± 27.8	137.4 ± 33.3
Triglycerides, mg/dL (*P* ≤ 0.0001)	85.1 ± 40.7	115.7 ± 67.6	99.4 ± 61.3	140.7 ± 88.1	121.0 ± 70.1	154.1 ± 106.9
Hematocrit, % (*P* ≤ 0.0001)	41.5 ± 3.7	42.5 ± 3.7	43.3 ± 3.5	43.6 ± 3.5	42.3 ± 2.8	42.9 ± 4.0
Red blood cell count, n/mm^3^ (*P* = 0.0001)	4.8 ± 0.5	4.9 ± 0.5	4.9 ± 0.5	5.1 ± 0.5	4.9 ± 0.5	4.9 ± 0.5
Hb, g/dL (*P* ≤ 0.0001)	14.0 ± 1.4	14.4 ± 1.4	14.6 ± 1.3	14.8 ± 1.3	14.2 ± 1.1	14.5 ± 1.5
White blood cell count, n/mm^3^ (*P* ≤ 0.0001)	6.4 ± 1.7	6.7 ± 1.7	6.7 ± 1.8	6.9 ± 1.7	7.7 ± 4.4	7.1 ± 1.6
Platelet count, n/mm^3^ (*P* ≤ 0.0001)	270 ± 63	266 ± 64	267 ± 67	259 ± 65	263 ± 53	250 ± 68
hsCRP, mg/L (*P* ≤ 0.0001)	1.7 ± 3.0	3.0 ± 4.1	2.3 ± 5.5	3.6 ± 5.0	1.1 ± 0.8	4.1 ± 4.4
hCys, µmol/L (*P* ≤ 0.0001)	13.3 ± 7.8	14.2 ± 9.7	14.6 ± 8.6	14.6 ± 8.2	12.0 ± 3.7	14.2 ± 5.5
hsCRP > 5 mg/L (*P* ≤ 0.0001)	65 (6.3%)	102 (13.5%)	15 (8.2%)	72 (18.1%)	0 (0%)	35 (24.5%)
Arterial hypertension# (*P* ≤ 0.0001)	140 (13.5%)	273 (36.2%)	68 (37.2%)	241 (60.5%)	12 (42.9%)	117 (81.8%)

Data are means ± SD or counts (percentage). BMI, body mass index; CA.ME.LI.A, CArdiovascular risks, MEtabolic syndrome, LIver and Autoimmune disease; DM, diabetes mellitus; hCys, hyperhomocysteinemia; hsCRP, high-sensitivity C‐reactive protein; IFG, impaired fasting glucose; NBW, normal body weight; NFG, normal fasting glucose; OWO, overweight-obese.

#Arterial hypertension was diagnosed in subjects who presented at least one of the following: systolic arterial pressure ≥140 mmHg, diastolic arterial pressure ≥90 mmHg, or treatment with antihypertensive therapy.

The distribution of MAFLD was different in the six subgroups. In NBW subjects, the percentage of subjects with MAFLD increased considerably from NFG to IFG to DM class (10.3% to 21% to 48%; *P* < 0.0001). In OWO/NFG subjects, the percentage with MAFLD was 50.3, increasing progressively to 68.4 in IFG/OWO and 82.9 in OWO/DM (*P* < 0.0001). Females had a lower percent incidence of MAFLD than males in the NFG/NBW (7.6% vs. 15.4%), NFG/OWO (46.7% vs. 54.2%), IFG/NBW (16% vs. 25.6%), and IFG/OWO (61.9% vs. 72.1%) groups, whereas they had a higher incidence in the DM/NBW (50% vs. 46.6%) and DM/OWO (88.1% vs. 79.4%) groups (Table 4). The thickness of visceral adipose tissue (VAT) progressively increased in male and female subjects in parallel with glucose and BMI increases. VAT was more represented in men than in women in the whole population as well as after dividing subjects according to fasting glucose levels or BMI (*P* ≤ 0.0001). Similar trends were observed in the distribution of the thickness of subcutaneous adipose tissue (SAT) in the entire population, with significantly more SAT in females as compared with males, in each of the six subgroups (Table 4 and Supplemental Fig. S3).

**Table 4.** Incidence of MAFLD and thickness of VAT and SAT in the population stratified by glucose tolerance and BMI

	NFG/NBW	NFG/OWO		IFG/NBW		IFG/OWO		DM/NBW		DM/OWO	
	Counts (%)	Counts (%)	χ^2^*	Counts (%)	χ^2^*	Counts (%)	χ^2^*	Counts (%)	χ^2^*	Counts (%)	χ^2^*
*n*	1,039; M = 372, F = 667	754; M = 395, F = 359		183; M = 105, F = 78		398; M = 269, F = 129		28; M = 15, F = 13		143; M = 95, F = 48	
MAFLD											
All	103 (10.3%)	324 (50.3%)	<0.0001	33 (21.0%)	0.0021	197 (68.4%)	<0.0001	12 (48.0%)	<0.0001	87 (82.9%)	<0.0001
Female	50 (7.6%)	156 (46.7%)	<0.0001	12 (16%)	0.0275	65 (61.9%)	<0.0001	6 (50.0%)	0.0003	37 (88.1%)	<0.0001
Male	53 (15.4%)	168 (54.2%)	<0.0001	21 (25.6%)	0.1694	132 (72.1%)	<0.0001	6 (46.1%)	0.0161	50 (79.4%)	<0.0001

	Means ± SD	Means ± SD	*P*§	Means ± SD	*P*§	Means ± SD	*P*§	Means ± SD	*P*§	Means ± SD	*P*§
VAT, mm										
All	22.8 ± 11.8	39.1 ± 17.3	<0.0001	28.1 ± 13.8	<0.0001	46.2 ± 20.4	<0.0001	30.8 ± 15.5	0.0197	54.0 ± 20.9	<0.0001
Female	19.8 ± 9.6 (64.2%)	34.2 ± 16.8 (47.6%)	<0.0001	21.2 ± 10.1 (42.6%)	0.9175	42.2 ± 20.8 (32.4)	<0.0001	27.5 ± 14.5 (46.4%)	0.2120	54.0 ± 20.1 (33.6%)	<0.0001
Male	28.2 ± 13.0 (35.8%)	43.5 ± 16.5 (52.4)	<0.0001	33.2 ± 13.9 (57.4%)	0.1694	48.0 ± 20.0 (67.6%)	<0.0001	33.5 ± 16.5 (53.4%)	0.4980	54.0 ± 21.4 (66.4%)	<0.0001
SAT, mm											
All	9.8 ± 5.8	16.4 ± 5.7	<0.0001	10.8 ± 4.1	0.1110	15.8 ± 6.1	<0.0001	8.1 ± 3.5	0.4692	15.6 ± 6.1	<0.0001
Female	10.2 ± 5.2 (64.2%)	18.3 ± 5.2 (47.6%)	<0.0001	11.1 ± 4.1 (42.6%)	0.9105	19.4 ± 5.4 (32.4)	<0.0001	9.5 ± 3.6 (46.4%)	0.9968	19.0 ± 5.8 (33.6%)	<0.0001
Male	9.2 ± 6.8 (35.8%)	14.8 ± 5.6 (52.4)	<0.0001	10.6 ± 4.1 (57.4%)	0.1448	14.1 ± 5.6 (67.6%)	<0.0001	6.9 ± 3.1 (53.4%)	0.5195	13.9 ± 5.6 (66.4%)	<0.0001

In males, echographic data are missing in 256 of 1,251 subjects, and in females, echographic data are missing in 71 of 1,294 subjects. DM, diabetes mellitus; IFG, impaired fasting glucose; MAFLD, metabolic (dysfunction)-associated fatty liver disease; NBW, normal body weight; NFG, normal fasting glucose; OWO, overweight-obese; SAT, subcutaneous adipose tissue; VAT, visceral adipose tissue.

*Chi-square test (Fisher’s exact test). The data (*P* value) represent the comparison of each group with the reference NFG/NBW group;

§One-way ANOVA (Šídák’s multiple-comparisons test). The data (*P* value) represent the comparison of each group with the reference NFG/NBW group.

### Association between Glucose Tolerance and/or Obesity with Death for All Causes

Mortality at the 7-yr follow-up of the CA.ME.LI.A study, by considering the stratification in the six subgroups, is reported in Table 5. A total of 80/2,545 (3.1%) apparently healthy subjects died. Among them, cancers accounted for 41.2% (*n* = 33) of all deaths, whereas cardio-cerebrovascular causes accounted for 17.5% (*n* = 14). Other diseases [20% (*n* = 16)] and unspecified causes [21.3% (*n* = 17)] accounted for the remaining deaths.

**Table 5.** Causes of death in subjects with different levels of glucose tolerance (normal, impaired, and diabetes) and body mass index (normal and overweight/obese)

Causes of Death	NFG/NBW	NFG/OWO	IFG/NBW	IFG/OWO	DM/NBW	DM/OWO
All causes (*n* = 80)	15/1,039 (1.44%)	16/754 (2.12%)	7/183 (3.8%)	19/398 (4.7%)	1/28 (3.5%)	22/143 (15.3%)
Cardio- and cerebrovascular (*n* = 14)	2/1,039 (0.19%)	4/754 (0.5%)	0	1/398 (0.25%)	0	7/143 (4.8%)
Cancer (*n* = 33)	6/1,039 (0.6%)	4/754 (0.5%)	5/183 (2.7%)	8/398 (2.0%)	0	10/143 (6.9%)
Other diseases (*n* = 16)	5/1,039 (0.6%)	1/754 (1%)	1/183 (1%)	4/398 (2.5%)	1/28 (3.5%)	4/143 (3.4%)
Unspecified cause (*n* = 17)	2/1,039 (0.2%)	7/754 (0.9%)	1/183 (0.6%)	6/398 (1.5%)	0	1/143 (0.7%)

DM, diabetes mellitus; IFG, impaired fasting glucose; NBW, normal body weight; NFG, normal fasting glucose; OWO, overweight-obese.

Mortality from all causes increased as plasma glucose levels increased and further increased, within the same glucose level class, in the presence of overweight/obesity. The increase in death from NFG/NBW to DM/OWO seemed to display an exponential trend, with an apparent 15-fold increase (Table 6).

**Table 6.** All-cause deaths’ HRs in the study population

Glucose Tolerance/BMI Category	No. of Deaths	Hazard Ratio	95% CI	*P*
Whole population				
NGT/NBW (*n* = 1,039)^a^	15	1		
NGT/OWO (*n* = 754)	16	1.45	0.72–2.93	0.302
IFG/NBW (*n* = 183)	7	2.70	1.10–6.62	0.030
IFG/OWO (*n* = 398)	19	3.38	1.72–6.66	<0.001
DM/NBW (*n* = 28)	1	2.49	0.33–19	0.377
DM/OWO (*n* = 143)	22	11.78	6.11–23	<0.001
All subjects (*n* = 2,545)	80			
Men				
NGT/NBW (*n* = 372)^a^	5	1		
NGT/OWO (*n* = 395)	10	1.84	0.265	0.265
IFG/NBW (*n* = 105)	2	1.43	0.28–7.35	0.671
IFG/OWO (*n* = 269)	17	4.85	1.78–13.15	0.002
DM/NBW (*n* = 15)	0	0		
DM/OWO (*n* = 95)	17	14.99	5.53–41	<0.001
All subjects (*n* = 1,257)	51			
Women				
NGT/NBW (*n* = 667)^a^	10	1		
NGT/OWO (*n* = 359)	6	1.10	0.40–3.02	0.858
IFG/NBW (*n* = 78)	5	4.41	1.51–12.92	0.007
IFG/OWO (*n* = 129)	2	1.04	0.23–4.73	0.963
DM/NBW (*n* = 13)	1	5.43	0.70–42	0.107
DM/OWO (*n* = 48)	5	7.37	2.52–21	<0.001
All subjects (*n* = 1,294)	29			

BMI, body mass index; CI, confidence interval; DM, diabetes mellitus; HR, hazard ratio; IFG, impaired fasting glucose; NBW, normal body weight; NFG, normal fasting glucose; OWO, overweight-obese.

^a^Reference category for hazard ratio and 95% CI calculations.

**Figure 1.**
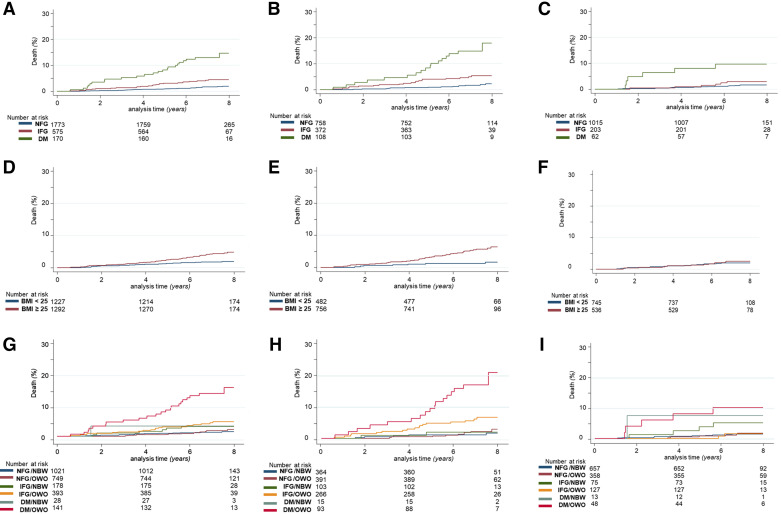
Incidence of deaths for all causes in the follow-up period of CA.ME.LI.A population according to—*A*: glucose tolerance in the whole population: NFG = 1.75%, IFG = 4.52%, DM = 13.53%; *B*: glucose tolerance in the male population: NFG = 1.9%, IFG = 5.11%, = DM 17.74%; *C*: glucose tolerance in the female population: NFG = 1.58%, IFG = 3.45%, DM = 9.68%; *D*: BMI in the whole population: BMI of <25 (NBW) = 1.87%, BMI of >25 (OWO) = 4.41%; *E*: BMI in the male population: BMI of <25 (NBW) = 1.45%, BMI of >25 (OWO) = 5.82%; *F*: BMI in the female population: BMI of <25 (NBW) = 2.15%, BMI of >25 (OWO) = 2.43% (In OWO, *P* = 0.003 between sexes); *G*: BMI and glucose tolerance in the whole population: NFG/NBW = 1.47%, NFG/OWO = 2.14%, DM/NBW = 3.57%, IFG/NBW = 3.93, IFG/OWO = 4.83%, DM/OWO = 15.6%; *H*: BMI and glucose tolerance in the male population: NFG/NBW = 1.37%, NFG/OWO = 2.56%, DM/NBW = 0%, IFG/NBW = 1.37%, IFG/OWO = 6.39%, DM/OWO = 18.28%; *I*: BMI and glucose tolerance in the female population: NFG/NBW = 1.52%, NFG/OWO = 1.68%, DM/NBW = 3.69%, IFG/NBW = 6.67, IFG/OWO = 1.57%, DM/OWO = 10.42%. BMI, body mass index; CA.ME.LI.A, CArdiovascular risks, MEtabolic syndrome, LIver and Autoimmune disease; DM, diabetes mellitus; IFG, impaired fasting glucose; NBW, normal body weight; NFG, normal fasting glucose; OWO, overweight-obese.

Figure 1 shows the Kaplan–Meier curves for the incidence of death from all causes according to glucose levels (panels *A*, *B*, and *C*), BMI (*D*, *E*, and *F*), and the combination of the two (*G*, *H*, and *I*). The effect of increased glucose levels on death rates is evident both when considering either the population as a whole or males and females separately. The increase in mortality of the IFG group compared with the NFG class is significant (*P* < 0.005) and similar between males and females. The increase in mortality of the DM group is significantly higher as compared with both NFG and IFG (both *P* < 0.0005) and more pronounced in males than in females (17.74% vs. 9.68%, *P* < 0.010) (Fig. 1, *A*–*C*, and Supplemental Fig. S4, *A*–*C*).

The effect of BMI on mortality, in contrast, shows sex-related differences. In the population as a whole, the increase in mortality in the OWO class as compared with the NBW class is significant (*P* < 0.0005). Interestingly, this increase is due to increased male mortality (*P* < 0.0005 in males), whereas in females, there is a nonsignificant difference between the two BMI classes (2.15% in NBW vs. 2.43% in OWO; *P* = nonsignificant) (Fig. 1, *D*–*F*, and Supplemental Fig. S4, *D*–*F*).

Considering the combined effects of glucose tolerance and BMI, when comparing the NFG/NBW with NFG/OWO, mortality was similar in males and females. Among females, however, having a BMI of <25 in the presence of IFG (IFG/NBW) resulted in increased mortality as compared with NFG/NBW and NFG/OWO (*P* < 0.005). In males, IFG/OWO showed increased mortality as compared with NFG/NBW and NFG/OWO (*P* < 0.0005). In both sexes, DM associated with NBW did not lead to a significant difference in mortality, possibly due to the low number of subjects in this category (*n* = 28). As expected, the DM/OWO class had the highest number of deaths in both males (18.3%) and females (10.4%) (*P* < 0.0005 in both) (Fig. 1, *G*–*I*, and Supplemental Fig. S4, *G*–*I*).

In Supplemental Table S4, odds ratio (OR) after comparison of mortality for all causes, for cardio-cerebrovascular causes, for cancer, and for all other causes, in each of the six classes versus the others (paired comparison), are reported. Chi-square (and Fisher’s exact) tests were performed to assess whether the discrepancy between the different classes was more than expected by chance. In particular, the OR (95% CI) of mortality was statistically significant (*P* < 0.0001) for malignant neoplasm and cardiovascular causes when DM/OWO was compared with NFG/NBW, NFG/OWO, and IFG/OWO and was significant for malignant neoplasm when DM/OWO was compared with IFG/NBW.

### Association of Glucose Tolerance and/or Obesity with Cardiovascular Events and Deaths

During the 7-yr follow-up period, 163 cardiovascular (CV) events (14 deaths from CV causes) occurred during an overall observation period of 17,776 person-years (Supplemental Table S5) The incidence rate of CV events in the whole population was 0.92 cases per 100 patient-years and was significantly higher in men, with an incidence rate ratio of 1.98 (95% CI: 1.42–2.78; *P* < 0.0001).

Supplemental Fig. S5*A* shows the Kaplan–Meier plot of the incidence of CV events and deaths among males and females (*P* < 0.0001). The two populations have also been analyzed separately (Supplemental Fig. 5, *B* and *C*, and Supplemental Table S6) to show the survival plots after the population was stratified into five age categories (≤35, 35–44, 45–54, 55–64, and ≥65 yr). In men, the incidence of CV events was consistently more frequent in all age categories, with a more marked difference with women in the oldest age-group (≥65 yr).

**Figure 2.**
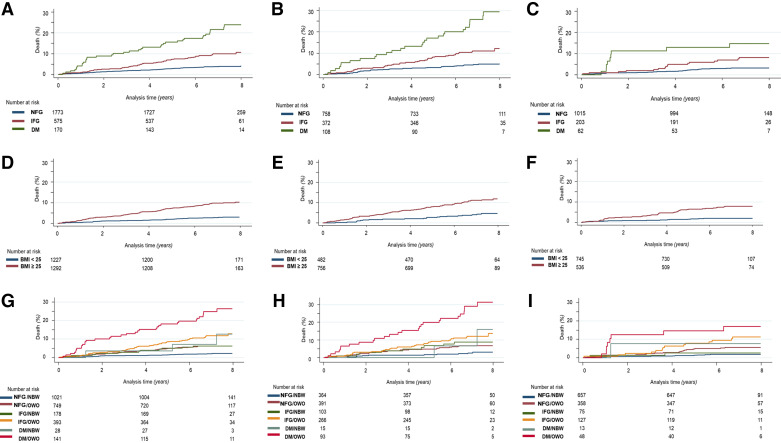
Incidence of cardiovascular (CV) events and death from CV causes in the follow-up period of CA.ME.LI.A population according to—*A*: glucose tolerance in the whole population: NFG = 3.84%, IFG = 9.74%, DM = 21.51%; *B*: glucose tolerance in the male population: NFG = 4.81%, IFG = 10.88%, DM = 25.45%; *C*: glucose tolerance in the female population: NFG = 3.12%, IFG = 7.69%, DM = 14.55%; *D*: BMI in the whole population: BMI of <25 (NBW) = 2.96%, BMI of >25 (OWO) = 9.66%; *E*: BMI in the male population: BMI of <25 (NBW) = 4.47%, BMI of >25 (OWO) = 10.98%; *F*: BMI in the female population: BMI of <25 (NBW) = 2.15%, BMI of >25 (OWO) = 7.8%; *G*: BMI and glucose tolerance in the whole population: NFG/NBW = 2.6%, NFG/OWO = 7.0%, DM/NBW = 10.70%, IFG/NBW = 7.5, IFG/OWO = 14.8%, DM/OWO = 24.0%; *H*: BMI and glucose tolerance in the male population: NFG/NBW = 1.37%, NFG/OWO = 3.76%, DM/NBW = 14.12%, IFG/NBW = 6.37%, IFG/OWO = 9.39%, DM/OWO = 30.28%; *I*: BMI and glucose tolerance in the female population: NFG/NBW = 1.80%, NFG/OWO = 5.57%, DM/NBW = 7.69%, IFG/NBW = 2.56, IFG/OWO = 10.85%, DM/OWO = 16.67%. BMI, body mass index; CA.ME.LI.A, CArdiovascular risks, MEtabolic syndrome, LIver and Autoimmune disease; DM, diabetes mellitus; IFG, impaired fasting glucose; NBW, normal body weight; NFG, normal fasting glucose; OWO, overweight-obese.

The incidence of CV events and deaths was significantly higher in the IFG and DM classes as compared with the NFG class and in DM as compared with IFG (all, *P* < 0.0005); the same trend was found in males and females but with higher values for all classes in males (Fig. 2, *A*–*C*; Supplemental Fig. S6, *A*–*C*, and Supplemental Table S7).

The same analysis was carried out by considering BMI, independently from glucose tolerance, comparing NBW versus OWO. Incidence of CV events and deaths according to BMI in the whole population was always higher in OWO than in NBW subjects (NBW: 2.96%; OWO: 9.66%), with similar trends in both females and males (*P* < 0.0001). However, there was a higher prevalence of events in men than in women in both BMI categories. Of interest is the observation that also in the NBW group, men had a prevalence of events more than twice higher than women (4.5% vs. 2.0%, *P* < 0.0001) (Fig. 2, *D*–*F*; Supplemental Fig. 6, *D*–*F*, and Supplemental Table S8).

The presence of impaired fasting glucose or diabetes increased CV events and mortality as compared with NFG, although to a different extent (DM > IFG). Similarly also OWO as compared with NBW showed a similar effect to increase CV events and deaths. Then we investigated the impact on cardiovascular events and deaths, when hyperglycemia (IFG or DM) was present along with overweight/obesity.

Table 7 reports the incidence of CV events and deaths in the population stratified into the six subgroups based on glucose tolerance and BMI categories. The Kaplan–Meier survival curves are shown in Fig. 2, *G*–*I*. In the whole population, the incidence of CV events and deaths increased progressively from about twice in NGT/OWO to more than 12-fold in DM/OWO as compared with the NFG/NBW group (Table 7). A similar trend toward worsening CV morbidity/mortality across the six glucose tolerance and BMI levels was present when men and women were analyzed separately. In addition, when sex was included in Cox modeling, the female sex emerged as a factor conditioning a more favorable CV prognosis (HR to men: 0.70; 95% CI: 0.50–0.97; *P* = 0.034).

**Table 7.** Cerebro-cardiovascular deaths and events’ HRs based on glucose tolerance and BMI during the follow-up

Glucose Tolerance/BMI Category	No. of CV Events	Hazard Ratio	95% CI	*P*
Whole population			
NFG/NBW (*n* = 1,039)^a^	23	1		
NFG/OWO (*n* = 754)	46	2.78	1.68–4.59	<0.001
IFG/NBW (*n* = 183)	11	2.81	1.37–5.77	0.005
IFG/OWO (*n* = 398)	46	5.51	3.34–9.08	<0.001
DM/NBW (*n* = 28)	3	4.88	1.47–16	0.010
DM/OWO (n = 143)	34	12.71	7.48–22	<0.001
All subjects (*n* = 2,545)	163			
Men				
NFG/NBW (*n* = 372)^a^	11	1		
NFG/OWO (*n* = 395)	26	2.25	1.11–4.54	0.025
IFG/NBW (*n* = 105)	9	2.97	1.23–7.18	0.015
IFG/OWO (*n* = 269)	32	4.27	2.15–8.47	<0.001
DM/NBW (*n* = 15)	2	4.41	0.97–20	0.053
DM/OWO (*n* = 95)	26	11.3	5.58–22	<0.001
All subjects (*n* = 1,257)	106			
Women				
NFG/NBW (*n* = 667)^a^	12	1		
NFG/OWO (*n* = 359)	20	3.10	1.52–6.35	0.002
IFG/NBW (*n* = 78)	2	1.49	0.33–8.67	0.599
IFG/OWO (*n* = 129)	14	6.23	2.88–13	<0.001
DM/NBW (*n* = 13)	1	4.40	0.47–34	0.155
DM/OWO (*n* = 48)	8	10.27	4.20–25	<0.001
All subjects (*n* = 1,294)	57			

CV events are first hospitalization for CV problems or deaths from CV cause. BMI, body mass index; CI, confidence interval; CV, cardiovascular; DM, diabetes mellitus; HR, hazard ratio; IFG, impaired fasting glucose; NBW, normal body weight; NFG, normal fasting glucose; OWO, overweight-obese.

^a^Reference category for hazard ratio and 95% CI calculations.

Figure 2, *G*–*I*, shows that the incidence of cardiovascular events and deaths was significantly lower in NFG/OWO versus DM/OWO subjects in the whole population (*P* < 0.0001), as well as in men (*P* ≤ 0.0001) and women (*P* = 0.002). Likewise also NFG/OWO had significantly less events as compared with IFG/OWO subjects in the whole population (*P* = 0.0009), in men (*P* = 0.01), and in women (*P* = 0.04). In women, there was a greater incidence of CV events in the IFG/OWO group (HR: 6.23 compared with NFG/NBW) than in men in the same group (HR: 4.27 compared with NFG/NBW). In men, the increase in CV events was similar in NFG/OWO and IFG/NBW groups (HR: 2.25 and 2.97, respectively), as compared with NFG/NBW, and in IFG/OWO and DM/NBW (HR: 4.27 and 4.41, respectively), as compared with NFG/NBW, with the highest increase being observed in the DM/OWO group (HR: 11.3). In women, an increase in glucose levels did not result in such a higher CV risk increase as in men, whereas a higher BMI resulted in a greater CV risk increase than in men. In women, NFG/OWO and IFG/NBW had significantly different CV risk increase (HR: 3.10 and 1.49, respectively), with a similar trend observed in IFG/OWO and DM/NBW groups (HR: 6.23 and 4.40, respectively). Finally, in women, the group at highest risk was the DM/OWO (HR: 10.27, compared with NFG/NBW) (Table 7).

### Univariate and Multivariate Analysis

A multivariate analysis was performed to assess whether overweight/obesity, impaired fasting glucose, and diabetes were significantly associated with cardiovascular deaths and events even after correction for other variables.

Before the multivariate analysis, univariate analyses were performed to understand the distribution of values for each variable of major interest. The variables used are continuous, when possible, or categorical. Only those found to be significant (*P* < 0.10 level) in the univariate Cox analyses have been included in the multivariate analysis (Table 7). Male and female populations were analyzed separately, as sex was significantly associated with most study variables.

In men, LDL cholesterol (*P* = 0.28) and SAT (*P* = 0.513) did not show statistically significant associations with cardiovascular events and are not reported. All variables significantly associated with the events in the univariate analyses are reported in Table 8 and were included in the multivariate analysis.

**Table 8.** Univariate and multivariate analysis of cardiovascular events and deaths versus glucose intolerance/obesity

Variables	General Population (*n* = 2,545)				Men (*n* = 1,251)				Women (*n* = 1,294)			
	Univariate Analysis	Multivariate Analysis			Univariate Analysis	Multivariate Analysis			Univariate Analysis	Multivariate Analysis		
	*P*	*P*	HR	95% CI	*P*	*P*	HR	95% CI	*P*	*P*	HR	95% CI
Age, yr	<0.0001	<0.0001	1.08	1.0–1.1	<0.0001	<0.0001	1.08	1.0–1.1	<0.0001	<0.0001	1.09	1.0–1.1
hsCRP elevated^a^, mg/L	<0.0001	0.002	1.90	1.3–2.9	<0.0001	<0.0001	3.06	1.8–5.3	ns	ns	ns	ns
HDL, mg/dL	<0.0001	<0.0001	0.97	0.93–0.98	0.003	0.041	0.97	0.9–1.0	ns	ns	ns	ns
LDL, mg/dL	0.013	0.251	1.00	0.99–1.00	ns	ns	ns	ns	0.001	0.681	1.00	1.0–1.1
Diabetes mellitus	<0.0001	<0.0001	3.51	2.4–5.1	<0.0001	0.025	2.02	1.1–3.2	ns	ns	ns	ns
Glucose intolerance (100–125 mg/dL)	<0.0001	<0.0001	2.13	1.5–3.1	<0.0001	0.001	2.11	1.4–3.3	<0.0001	0.052	1.84	1.0–3.4
BMI, kg/m^2^	<0.0001	<0.0001	2.78	1.9–4.1	<0.0001	0.7	0.98	0.9–1.1	ns	ns	ns	ns
Triglycerides, mg/dL	<0.0001	0.375	1.00	0.99–1.00	0.001	0.224	1.00	0.99–1.0	0.003	0.518	1.00	1.0–1.1
Hypertension	<0.0001	0.083	1.52	0.95–2.44	<0.0001	0.130	1.59	0.9–2.9	<0.0001	0.311	1.48	0.7–3.1
Metabolic syndrome	<0.0001	0.250	0.75	0.46–1.22	<0.0001	0.114	0.57	0.3–1.1	<0.0001	0.672	0.85	0.4–1.8
HOMA index	<0.0001	0.095	1.06	0.99–1.14	<0.0001	0.068	1.07	1.0–1.2	<0.0001	0.702	1.02	0.9–1.2
MAFLD	<0.0001	0.758	1.06	0.71–1.59	0.006	0.735	1.09	0.7–1.8	<0.0001	0.442	1.27	0.7–2.4
VAT elevated^b^	0.0018	0.367	0.80	0.49–1.30	0.005	0.517	1.25	0.6–2.5	ns	ns	ns	ns

BMI, body mass index; CI, confidence interval; CRP, C-reactive protein; HR, hazard ratio; hsCRP, high-sensitivity C-reactive protein; MAFLD, metabolic (dysfunction)-associated fatty liver disease; ns, not significant; VAT, visceral adipose tissue.

^a^Values above the upper reference limit for CRP (>5 mg/L).

^b^Values in the three upper quartiles of the VAT distribution (Q2, Q3, Q4) >16 mm.

**Figure 3.**
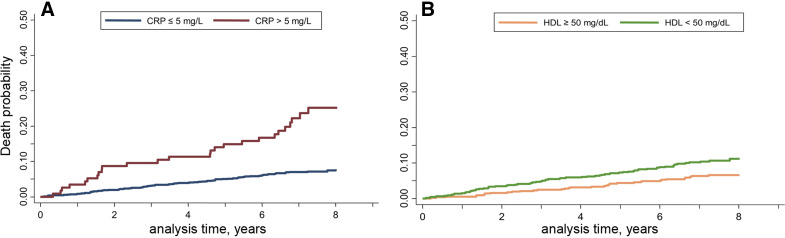
Incidence of cardiovascular events in the male CA.ME.LI.A population stratified by CRP values (*A*, cutoff = 5 mg/dL) and HDL values (*B*, cutoff = 40 mg/dL). CA.ME.LI.A, CArdiovascular risks, MEtabolic syndrome, LIver and Autoimmune disease; CRP, C-reactive protein.

In men, only age (<0.0001), the presence of elevated CRP levels (<0.0001), HDL cholesterol (*P* = 0.03), diabetes (*P* = 0.02), and BMI retained a statistically significant association with CV events after correction for the other variables. Figure 3*A* shows the survival curves of men according to high-sensitivity CRP, where death probability is higher with CRP values above the cutoff (*P* < 0.0001). Figure 3*B* shows survival plots based on HDL cholesterol, highlighting a significantly higher incidence of CV events in subjects with levels under the 40 mg/dL cutoff (*P* = 0.020).

In contrast, CRP (*P* = 0.122), HDL (*P* = 0.086), and high VAT (*P* = 0.46) and SAT (*P* = 0.116) were not included in Table 8, for females, as they did not show a statistically significant association with cardiovascular events. All the other variables with a significant association with CV events have been included in the table. Multivariate analysis shows that all the variables lost their significant effect after correction for the other factors, with the exception of age, which maintains its high level of statistical significance (<0.0001).

## DISCUSSION

Overweight and obesity have reached epidemic proportions around the world, affecting almost 60% of adults and one in three school-aged children (33, 34). The data collected on the prevalence of obesity in the study population are consistent with the ISTAT (Italian National Institute of Statistics) data collected in the Italian population during that period showing a prevalence of obesity and overweight of 45.9% (consistent with this reported prevalence, in our study population it was 51.1%), of which 37.0% in females and 55.6% in males (in our study population, females were 41.6% and males were 60.9%) (35). Regarding the prevalence of diabetes mellitus (type 1 and type 2) found by ISTAT in the Italian general population during the study period, this accounted for 5.3% (5.4% for males and 5.2% for females) (36); our study population showed comparable figures for the total prevalence of diabetes (type 1 and type 2: 6.7%), with greater intersex variability (8.7% for males and 4.8% for females). In addition, 22.9% had impaired fasting glucose, i.e., fasting blood sugar levels between 100 and 125 mg/dL. The sum of the two percentages (29.6%) shows that almost one-third of the general population in this study had altered glucose levels (prediabetes or diabetes). Of note, in the ISTAT database, the incidence of impaired fasting glucose (prediabetes) is not present (36).

Sex impacts the pathogenesis of numerous diseases, including metabolic disorders such as diabetes. In most parts of the world, diabetes is more prevalent in men than in women, especially in middle-aged populations (37). In our study population, men with IFG and men with DM were about double than women in the same categories (30.0% and 8.7% vs. 16.0% and 4.8%). Sex-specific differences have been found in glucose homeostasis, insulin secretion and action, and the incidence and progression of diabetes, which might help explain this different distribution (38). Only in people aged ≥65, there were more women with DM than men, and this was probably due to women’s greater longevity (39).

With increasing age, worsening of glucose tolerance (IFG and DM) emerged, confirming the importance of advanced age as a risk factor for glucose intolerance, independently from BMI (40). IFG and DM subjects had a significantly higher BMI than NFG subjects, especially in men. This observation is consistent with overweight/obesity being an independent and dose-dependent risk factor for type 2 diabetes mellitus (40).

Overweight/obesity also correlated with an increased thickness of VAT and SAT as compared with NBW. VAT was increased in subjects with impaired fasting glucose and diabetes as compared with NFG subjects, both in women and men, although VAT was thicker in men as compared with women. VAT increased significantly in size in the diabetic and intolerant population in both men and women. SAT is always higher in OWO independently from glucose tolerance and higher in diabetic women than diabetic men, possibly confirming that women start already with larger stores of subcutaneous fat, whereas men present more visceral fat (41). Both SAT and VAT are associated with increased subinflammation and oxidative stress (42). Definitely, VAT has a stronger role compared with SAT in the onset of insulin resistance and diabetes, beyond the increased release of free fatty acid and possibly due to increased levels of proinflammatory cytokines (16, 21, 22, 43). Obesity is in fact the most important risk factor for type 2 diabetes, and they share a number of clinical manifestations, including metabolic syndrome X of insulin resistance, MAFLD, dyslipidemia, and arterial hypertension, which markedly increase morbidity and mortality (18, 24, 25, 44). VAT, SAT, and MAFLD showed similar trends to changes with BMI and fasting blood glucose, confirming their intimate connection (26, 31, 32, 45–54).

Aging is also associated with increased number cardiovascular events and deaths, as they were associated also to biological decline, and, as expected, the mortality became higher with increasing age. Nonetheless, at the same age, there were significant differences between the two sexes, with men having more cardiovascular events and deaths than women; this difference in life expectancy and mortality is in line with data reported in literature (55, 56). In both men and women, the risks associated with CVD increase with age, and these correspond to an overall decline in sex hormones, primarily estrogen and testosterone (57). In either sex, in the same-age groups, higher mortality was seen in the groups with OWO, IFG, and DM than in those with NBW or NFG.

Obesity, even when not associated with metabolic derangements, is a risk factor for the incidence of cardiovascular events and/or premature death (3, 6). In fact, in the NFG/OWO group, there was a statistically higher incidence of events than the NFG/NBW group. Cardiovascular disease risk is increased in populations with overweight and obesity classified as metabolically healthy, even in the absence of metabolic risk factors (58). This discrepancy in the incidence of cardiovascular events in men and women, even at high BMI, had already been reported in the literature: a high BMI has been shown to be more predictive of death from cardiovascular diseases, more in men than in women (3), and in women, a high fat content, regardless of the level of muscle mass, appears to be associated with a lower risk of mortality from cardiovascular diseases (59). Important differences between men and women are present also in the prescription, adherence, and response to cardiovascular drugs (60).

The group of diabetic and normal body weight (DM/NBW) subjects showed a higher incidence of cardiovascular events than the overweight/obese normal glucose-tolerant (NFG/OWO) group, whereas DM/NBW and IFG/OWO had similar incidence of events. Among individuals with or without diabetes, absolute rates of cardiovascular disease are higher in men than in women; however, in reproductive-age women, the presence of type 2 diabetes largely eliminates this protection from cardiovascular disease, and diabetic women are more severely affected than men (61, 62). Interestingly, in males, the DM/NBW group had a higher incidence of events than the IFG/OWO group, whereas in females, it was the opposite. These findings seem to imply that the factors weighing the most on the risk of cardiovascular events and deaths, differed by sex, were hyperglycemia (any value higher that 100 mg/dL) for males and overweight/obesity (any BMI value over 25 kg/m^2^) for females. Further studies are needed to validate this observation. This observation could also be partially explained by the fact that although IFG and DM subjects generally show a higher BMI than NFG subjects, this increase is more pronounced in men than in women. Pre-DM and undiagnosed T2DM play a more prominent role in the risk of CVD in women than in men. Women must experience a greater overall metabolic deterioration; that is, they must accumulate more fat and experience greater insulin resistance and related risk factors to evolve from normoglycemia to T2DM. Women’s higher diabetic CVD risk than that of men could be ascribed to their greater and more prolonged decline in metabolic homeostasis during the pre-DM stage (63–65).

Regarding anthropometric and clinical parameters changes, subjects in the IFG/OWO and DM/OWO groups showed higher levels of blood pressure, waist circumference, glycemia, cholesterol, and triglycerides as compared with NFG/NBW and NFG/OWO subjects (44, 66, 67). The HOMA index, within the same glucose tolerance class, was higher in OWO subjects than in NBW subjects. Men and women had comparable values, except in diabetic subjects, where NBW women had lower values than NBW men, whereas OWO women had higher values than OWO men. A recent study showed higher HOMA-IR cutoff values for women (68). Differences in insulin sensitivity between men and women may be due to differences in adipose tissue, muscle mass, hormones, and body fat distribution (subcutaneous and visceral fat) (38, 69). High-sensitivity C-reactive protein (hsCRP) increased as glucose intolerance increased, and, within different glucose tolerance classes, it was significantly greater in OWO subjects than in NBW subjects and in women compared with men. Systemic subinflammation has been extensively demonstrated to be strongly related to the patient’s metabolic state and lipid profile. Increased levels of inflammation markers and cytokines have also been associated with a higher risk of developing CVD, especially in women (70). In an analysis of patients from the Framingham Heart Study, the expression of biomarkers representing inflammatory pathways, including CRP, was seen to be higher in women (71). The risk of both macro- and microvascular complications increases before glucose levels reach the diagnostic threshold of diabetes, and 25% of newly diagnosed diabetics already have manifested cardiovascular disease (72–77). Our data underline the possibility of preserving long-term health in people with IFG before type 2 diabetes becomes established. In fact IFG/NBW, DM/NBW, IFG/OWO, and DM/OWO as compared with NFG/NBW display a progressive exponential increase of mortality for all causes, with ORs of 2.7, 2.5, 3.4, and 12.4, respectively, as compared with NFG/NBW.

Therefore, although there is over 10-fold increase in mortality in DM/OWO, consistent with previous studies, also in prediabetic individuals (IFG/NBW and IFG/OWO), there is an approximately threefold increase in mortality for all causes (cancer, cardiovascular, other disease, and unspecified causes). This suggests that aggressively treating IFG and OWO with diet, exercise, and possibly also medications, if necessary, might lower risk of cancer, cardiovascular events, and deaths (78, 79).

The efficacy of lowering blood pressure and blood glucose and lipid-lowering therapies has been shown to reduce subsequent morbidity and mortality in patients with diabetes and without diabetes (80–82), with no different beneficial preventive effects on the incidence of type 2 diabetes and weight gain between men and women (83). Interventions to improve lifestyle are necessary well before the onset of these diseases, and the maintenance of a healthy BMI and normal blood glucose, lipids, and pressure should be recommended in the general population to prolong healthy life.

### Limitations

Because the observations referred to the participants’ baseline data (clinical, laboratory, and echography), any changes in parameters during follow-up could not be considered. Approximately 20% of the deaths lacked identifiable causes, and deaths categorized as “unspecified other causes” might not account for potential cardiovascular events contributing to mortality. These limitations could result in an underestimation of deaths for cardiovascular causes or misclassification of events due to changes in participants’ weight or glucose tolerance status over time.

### Conclusions

Impaired fasting glucose, diabetes, and overweight/obesity increase the risk of cardiovascular events and all-cause mortality, both independently and more so when present in combination. Any worsening of glucose tolerance has a proportional effect in increasing mortality and the incidence of cardiovascular events. The individual contribution of each condition is at least, in part, sex-dependent, since in women, obesity causes the greatest increase in incidence, whereas in men, this is caused by diabetes.

## DATA AVAILABILITY

Data will be made available upon reasonable request.

## SUPPLEMENTAL MATERIAL

10.7910/DVN/L7TFFK Supplemental Figs. S1–S6 and Supplemental Tables S1–S8: https://doi.org/10.7910/DVN/L7TFFK.

## GRANTS

The CA.ME.LI.A project was supported by Regione Lombardia (DG Sanità 08/07/2008 n. 7364), Italian Ministry for Education (MIUR, GR-2011 02350447), and by Dipartimento di Scienze della Salute, Università degli Studi di Milano (LINEA 2 DEL PIANO DI SOSTEGNO ALLA RICERCA PSR2020_DIP_013_PARONI dal titolo: Looking for Novel Biomarkers of Diabetes and Cardiovascular Diseases, “NODICARDIO”). The study was approved by the Ethical Committee of Ospedale San Paolo. L.C. is a PhD student in “Scienze della nutrizione - Università degli Studi di Milano” supported by Project PNC 0000001 D3 4 Health—CUP [B83C22006120001], The National Plan for Complementary Investments to the NRRP, funded by the European Union—NextGenerationEU.

## DISCLOSURES

No conflicts of interest, financial or otherwise, are declared by the authors.

## AUTHOR CONTRIBUTIONS

R.P., P.M.B., and F.F. conceived and designed research; P.Z. and E. Bertolini performed experiments; M.B., E. Bianco, L.C., A.R., M.D.C., P.Z., C.M., C.B., M.Z., R.P., P.M.B., and F.F. analyzed data; F.B., P.M.B., and F.F. interpreted results of experiments; L.C., M.D.C., C.M., F.S., and P.M.B. prepared figures; M.B., E. Bianco, L.C., M.D.C., R.P., and F.F. drafted manuscript; L.C., R.P., and F.F. edited and revised manuscript; R.P. and F.F. approved final version of manuscript.
